# Red Light Combined with Blue Light Irradiation Regulates Proliferation and Apoptosis in Skin Keratinocytes in Combination with Low Concentrations of Curcumin

**DOI:** 10.1371/journal.pone.0138754

**Published:** 2015-09-18

**Authors:** Tianhui Niu, Yan Tian, Qing Cai, Qu Ren, Lizhao Wei

**Affiliations:** 1 Aviation Medicine Research Laboratory, The General Hospital of the Air Force, Beijing, China; 2 Department of Dermatology, The General Hospital of the Air Force, Beijing, China; 3 Department of Clinical Examination, The General Hospital of the Air Force, Beijing, China; University of Alabama at Birmingham, UNITED STATES

## Abstract

Curcumin is a widely known natural phytochemical from plant *Curcuma longa*. In recent years, curcumin has received increasing attention because of its capability to induce apoptosis and inhibit cell proliferation as well as its anti-inflammatory properties in different cancer cells. However, the therapeutic benefits of curcumin are severely hampered due to its particularly low absorption via trans-dermal or oral bioavailability. Phototherapy with visible light is gaining more and more support in dermatological therapy. Red light is part of the visible light spectrum, which is able to deeply penetrate the skin to about 6 mm, and directly affect the fibroblast of the skin dermis. Blue light is UV-free irradiation which is fit for treating chronic inflammation diseases. In this study, we show that curcumin at low concentrations (1.25–3.12 μM) has a strong anti-proliferative effect on TNF-α-induced psoriasis-like inflammation when applied in combination with light-emitting-diode devices. The treatment was especially effective when LED blue light at 405 nm was combined with red light at 630 or 660 nm, which markedly amplified the anti-proliferative and apoptosis-inducing effects of curcumin. The experimental results demonstrated that this treatment reduced the viability of human skin keratinocytes, decreased cell proliferation, induced apoptosis, inhibited NF-κB activity and activated caspase-8 and caspase-9 while preserving the cell membrane integrity. Moreover, the combined treatment also down-regulated the phosphorylation level of Akt and ERK. Taken together, our results indicated that the combination of curcumin with LED blue light united red light irradiation can attain a higher efficiency of regulating proliferation and apoptosis in skin keratinocytes.

## Introduction

In recent years, phototherapy with visible light is gaining increasing attention in dermatological practice. The Light-emitting diodes (LEDs), possessing a very narrow bandwidth, can distribute their biological effects to the defined wavelengths. [1]. Red light (wavelength range from 620 nm to 770 nm), which is part of the visible light spectrum, is able to directly impact the fibroblast of the skin dermis due to its capability to deeply penetrate the skin to about 6 mm [2], thus it is favoured in photodynamic therapy (PDT). Blue light (wavelength range from 400 nm to 480 nm) is UV-free irradiation that shows fewer harmful side effects to mammalian cells than ultraviolet irradiation [3]. Because of the effectiveness in reducing cell proliferation, blue light is propitious to treat hyperplastic diseases and chronic skin inflammation, such as psoriasis, atopic dermatitis and hand- and foot-eczema [4, 5]. It has been demonstrated that compared with UV light, irradiation with blue light at 400–420 nm only reveals toxic effects at high or very high dosages [6, 7]. The main biological effect of PDT is photochemical effect rather than thermal action [7,8].

Taking advantage of a photosensitizer in PDT can trigger different cellular reactions [9, 10]. Curcumin is a natural active photochemical composition of turmeric. Similar to resveratrol, it has shown antioxidant, anti-inflammatory, anti-carcinogenic and anti-microbial properties [10–12]. Moreover, curcumin has a rather wider absorption peak range from 300 nm to 500 nm [13]. Studies have shown that the maximum light absorption peak of curcumin is at about 420 nm [14]. However, the therapeutic activity of curcumin is hampered by its poor biological availability, which appears due to low absorption, fast metabolism and rapid systemic elimination [15, 16]. Most of studies show that curcumin induces apoptosis and suppresses cell proliferation in different cell lines at concentrations range from 10 μM to 150 μM. Research has proved that the effects of low dosages of curcumin can be strengthened through combination with visible light or UVA irradiation because the light energy intake is enhanced under these circumstances [14, 17].

Psoriasis is one of the most familiar immune system regulated chronic inflammatory skin disease, which is characterized by hyper-proliferation and abnormal differentiation of keratinocytes [18]. Tumour necrosis factor (TNF)-α is a critical pro-inflammatory cytokine in psoriasis immunopathology, over-expression of TNF-α is vital in pathogenesis of psoriasis, and suppression of TNF-α pathway is a key step in the regulation of psoriasis [19–21].

In this study we investigated whether the red light united blue light irradiation, in combination with low concentrations of curcumin, could efficiently attenuate TNF-α-induced dermatitis, which analogous to human psoriasis lesions [20].

## Materials and Methods

### Materials

Recombinant human TNF-α (Sino Biological Inc., China); curcumin (Sigma-Aldrich, St. Louis, MO, USA); pre-stained protein standards (Fermentas, Lithuania); Human Annexin V Apoptosis Detection Kit and cell cycle test kits (BD Biosciences, San Jose, CA); Bradford Assay kit (BD Biosciences, San Jose, CA). Antibodies against NF-κB-p65, phospho-NF-κB-p65, caspase-8, caspase-9, ERK, phospho-ERK (pERK), Akt andphospho-Akt (pAkt) (Cell Signaling Technology, Boston, MA, USA); β-actin antibody (Santa Cruz Biotechnology, USA); Nuclear and Cytoplasmic Protein Extraction Kit, LDH Cytotoxicity Assay Kit and CCK-8 Assay Kit (Beyotime, CHN); ECL detection kit (Cell Signaling Technology, Boston, MA, USA). Water was ultra-pured by a Milli-Q water purification system (Millipore, USA).

### Light source

**Fig 1:**
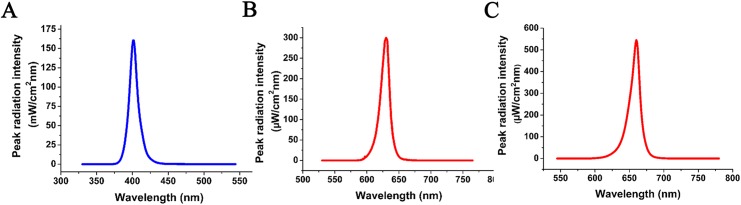
Detection map of LED light output intensity. (A) The maximum wavelength of blue light is at 405 nm. (B) The peak of R630 wavelength is at 630 nm. (C) The highest peak of R660 is at 660 nm.

The Light source was provided by the Chinese Semiconductor Research Institute. The output of light source was inspected at the Chinese National Institute of Metrology (Fig 1). The irradiation parameters were: blue LED illumination with a maximum intensity at 405 nm (161 μW/cm^2^nm), 10 min of application, cumulative dose 1.604 J/cm^2^; red LED illumination with a maximum strength at 630 nm (300 μW/cm^2^nm), 10 min of application, cumulative dose 3.409 J/cm^2^; red LED illumination with a maximum intensity at 660 nm (545 μW/cm^2^nm), 10 min of application, cumulative dose 6.538 J/cm^2^.

### Cell culture

HaCaT cells, the Human keratinocyte cell line, were obtained from Lifeline (Lifeline Cell Technology manufacture). HaCaT cells were cultured in Keratinocyte-SFM medium supplemented with growth factors (Life Factor) and 1% P/S solution. Cells were seeded in 60 mm dishes and passaged by treatment with trypsin/EDTA (Gibco). Cells were cultured at 37°C in a humidified atmosphere containing 5% CO_2_.

### Cell treatment

Curcumin was first dissolved in DMSO (20 mM/ml) and then diluted to the working concentrations with culture medium just before use. HaCaT cells were pre-incubated with curcumin containing medium for 2 h and then irradiated at a distance of 25 mm from the LED array. Ahead of irradiation, we replaced the medium with PBS to avoid the formation of reactive photochemical products within the culture medium. Cells were then irradiated with LED blue light, red light or a combination of blue and red light. After irradiation, cells were cultured with fresh medium. As non-irradiated controls, cells were kept in the dark condition during the irradiation course, in order to ensure the same processing conditions.

The cells were divided into ten groups: 1) the control group; 2) the curcumin group, which was only treated with curcumin but protected from light; 3) the red light group1, which was treated with red light (630 nm) alone; 4) the red light group2, which was treated with red light (660 nm) alone; 5) the blue light group, which was treated with blue light alone; 6) the red light added curcumin group 1, which was treated with curcumin and red light (630 nm) irradiation; 7) the red light added curcumin group 2, which was treated with curcumin and red light (660 nm) irradiation; 8) the blue light added curcumin group, which was treated with curcumin and blue light irradiation; 9) the blue united red light group 1, which was treated with curcumin and blue united red light (630 nm) irradiation; and 10) the blue added red group 2, which was treated with curcumin and blue united red light (660 nm) irradiation.

### Cell counting kit-8 assay

Cell viability was evaluated by CCK-8 assay as described previously [2]. Cells were seeded in 96-well plates at a density of 8 × 10^3^ cells/well. Different seeding densities had been optimized at the beginning of the experiments. Twenty hours later, cells were pre-incubated with curcumin (0.16–5 μM) for 2 h and then irradiated with LED blue light, red light and combined blue and red light as described above. At the end of the culture period, CCK-8 was added to each well according to the instructions and incubated at 37°C for 2 h. The absorbance was measured with a microplate reader (Spectra Max 190; Molecular Devices, Sunnyvale, CA) at a recording wavelength of 450 nm with a reference wavelength of 630 nm. The cell viability was shown as a percentum of control. Experiments were repeated for three times.

### Cytotoxicity

Cell lysis and cell death were quantified with a LDH Cytotoxicity Assay Kit (Beyotime). In brief, HaCaT cells were seeded in 96-well plates at a density of 6 × 10^3^ cells/well. Different seeding densities were optimized at the beginning of the experiments. Twenty hours later, cells were treated with curcumin (0.16–5 μM) and light irradiation as described above. After 20 h of treatment, the cell-free supernatants with incubated with NAD^+^, and positive controls were dealt with 1% Triton-X-100. Consequently, the NAD^+^ solution was reduced to NADH/H^+^ during the lactate dehydrogenase reaction, which altered the yellow tetrazolium salt to a red-coloured formazan salt. The absorbance was measured using an ELISA reader at 490 nm with a reference wavelength of 630 nm (Spectra Max 190; Molecular Devices, Sunnyvale, CA).

### Western blot analysis

Total proteins and nuclear extracts were isolated by the Nuclear and Cytoplasmic Protein Extraction Kit according to the manufacturer’s instruction. Cells were seeded in 60 mm dishes with a density of approximately 3 × 10^5^ cells/well for western blot tests. The protein concentrations were determined using the Bradford Assay kit (Bio-Rad) and the cell lysates were boiled with 5 × SDS gel-loading dye for 10 min at 100°C. The samples (20 μg/lane) were electrophoresed on 12% SDS-PAGE gel and transferred onto a polyvinylidene fluoride (PVDF) membrane (Millipore, Bedford, MA). After blocked with 5% non-fat milk in TBST buffer (0.1% Tween-20) for 1 h at room temperature, the membranes were incubated with the primary antibodies of interest at 4°C overnight. After washed the membranes three times with TBST, the membranes were exposed to horseradish peroxidase conjugated secondary antibodies for 2 h at room temperature. After washed the membranes three times with TBST, proteins bands were visualized by an ECL detection kit following the manufacturer’s instructions.

### Flow cytometry analysis

The cell cycle distribution was evaluated by flow cytometric analysis using cell cycle test kit (Becton Dickinson, San Jose, CA, USA). Cells were seeded in 35-mm plates (2 x 10^5^) and treated 20 h after seeding that at the confluence of about 70–80% as described above. For prior treatment, cells have been synchronized through starving in the basic medium without serum for more than sixteen hours in order to stop the cells in the same cycle/G0. After treatment, cells were collected by trypsinization, washed with PBS, resuspended in the solution A (trypsin buffer), incubated light-avoided for 10 min at room temperature, then successively added solution B (trypsin inhibitor and RNase buffer) and C (propidium iodide stain solution) according to the manufacturer’s protocol. The proportion of cells in G0/G1, S, and G2/M phases was represented as DNA histograms. For each test, we collected 1 x 10^4^ cells per specimen.

The apoptotic rate was surveilled by flow cytometric analysis using AV-FITC kit detection kit (Becton Dickinson, San Jose, CA, USA) according to the manufacturer’s protocol. Cells were plated in 35-mm plates (2 × 10^5^) and treated as described above. After treatment, cells were collected by trypsinization, washed twice with PBS, resuspended cells in 1 × binding buffer, and stained with 5 μL FITC Annexin V for 15 min in darkness. Finally, 5 μL propidium iodide (PI) dye was added just before testing. The apoptotic rate of cells were determined as the percentage of early apoptotic cells added late apoptotic cells. For each experiment 1 × 10^4^ cells per sample were collected.

All of the data were analyzed with the Cell Quest software (Becton Dickinson, CA, USA).

### Statistics

Data were presented as the mean±standard deviation. All average values were denoted as a percentum of the untreated control. Statistical analysis of data was performed using either Student's *t*-test or two-way analysis of variance (ANOVA) with Graph-Pad prism 5. Difference with *p*<0.05(*) or *p*<0.01(**) was considered statistically significant. All experiments were repeated at least three times.

## Results

### Curcumin combined with red united blue light inhibited cell viability

**Fig 2:**
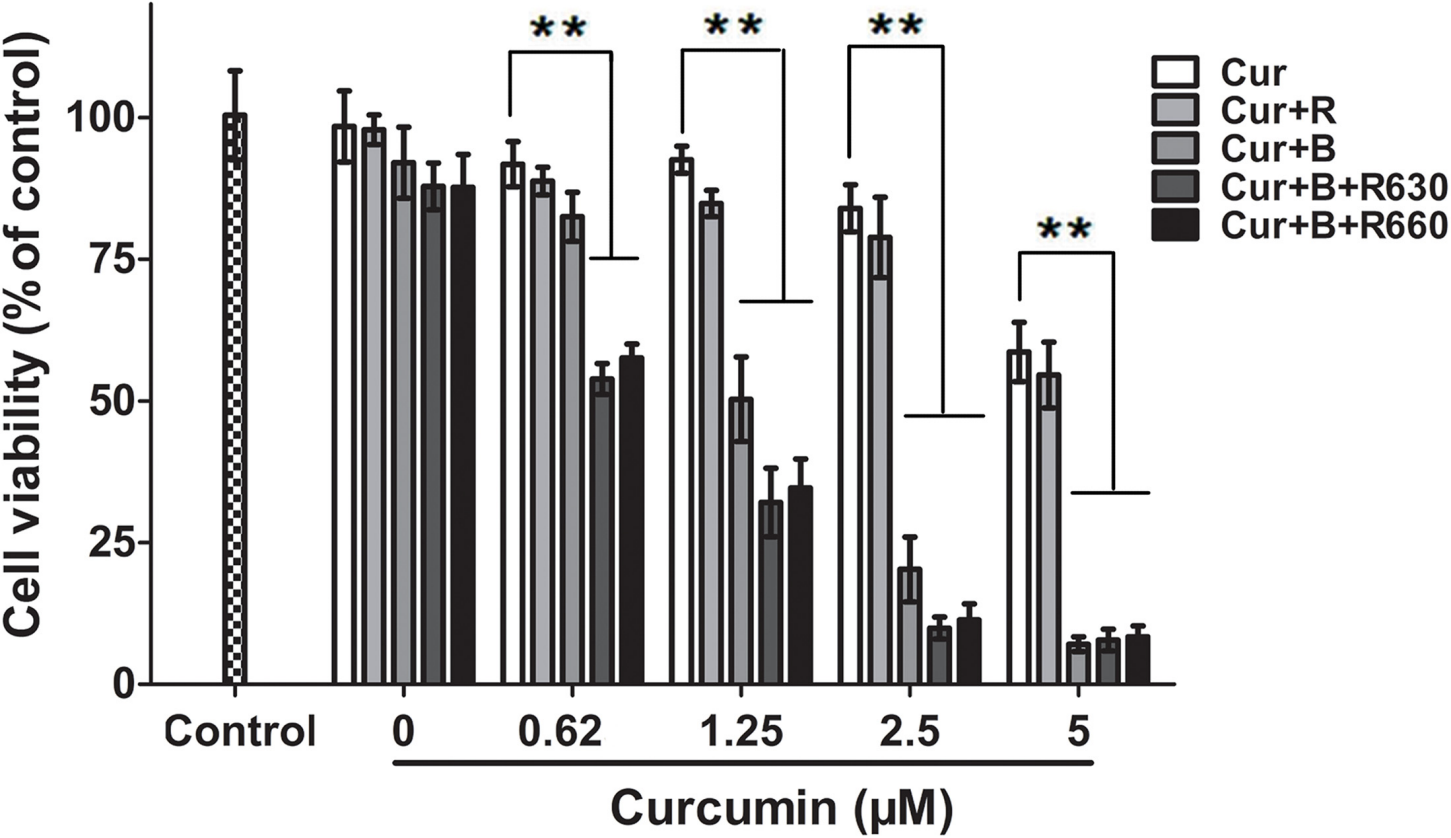
Curcumin combined with red united blue light irradiation restrained cell viability. HaCaT cells were pre-treated with curcumin (0–5 μM) for 2 h, and then separately irradiated with blue light, red light and two combinations of blue and red light, or protected from light. Cells without any treatment were used as a control. Cell viability was examined by CCK-8 assay 20 h after the last treatment. The values of the control were set to 100%. Each bar represents the mean of three independent experiments. The suppression rate is depended on the concentration of curcumin, and the differences between cells treated with light irradiation or not are particularly evident at *p*<0.05(*) or *p*<0.01(**) level.

To examine whether the treatment of curcumin combined with red united blue light could affect cell proliferation rate, we test the cell viability of HaCaT cells which were pre-incubated with curcumin (0–5 μM) for 2 h and then irradiated with blue light, red light or blue light combined with red light for 20 h. The data showed that both curcumin alone treated cells and single red or blue light irradiated cells showed tiny differences in cell viability. Exposure of curcumin-treated cells (1.25–5 μM) to blue light resulted in inhibition of HaCaT cell viability (*p<*0.05), especially when the irradiation was conducted with a combination of blue light and red light, which gave rise to a more distinct inhibition of cell proliferation (*p<*0.01) (Fig 2). At first, we irradiated curcumin-treated cells with blue light or red light for 5–15 minutes to determine the optimum conditions. The suppression rate was depended on the concentration of curcumin. In the curcumin-treated but light-protected controls, no obvious cell proliferation inhibition effects were detected in the concentrations from 0.16 μM to 2.5 μM, and weaker effects were found in the concentrations between 2.5 μM to 5 μM (*p*>0.05). The first noticeable change in density from the blue united red light was 0.16 μM, whereas the first appeared change from blue light alone was 0.62 μM (shown in S1 Fig). The single red light or blue light irradiated groups nearly showed no effect on the inhibition of cell proliferation (shown in Fig 2). These results certified that both curcumin and photo-activation were indispensable when using curcumin at low concentrations.

### Curcumin associated with red united blue light preserved membrane integrity

**Fig 3:**
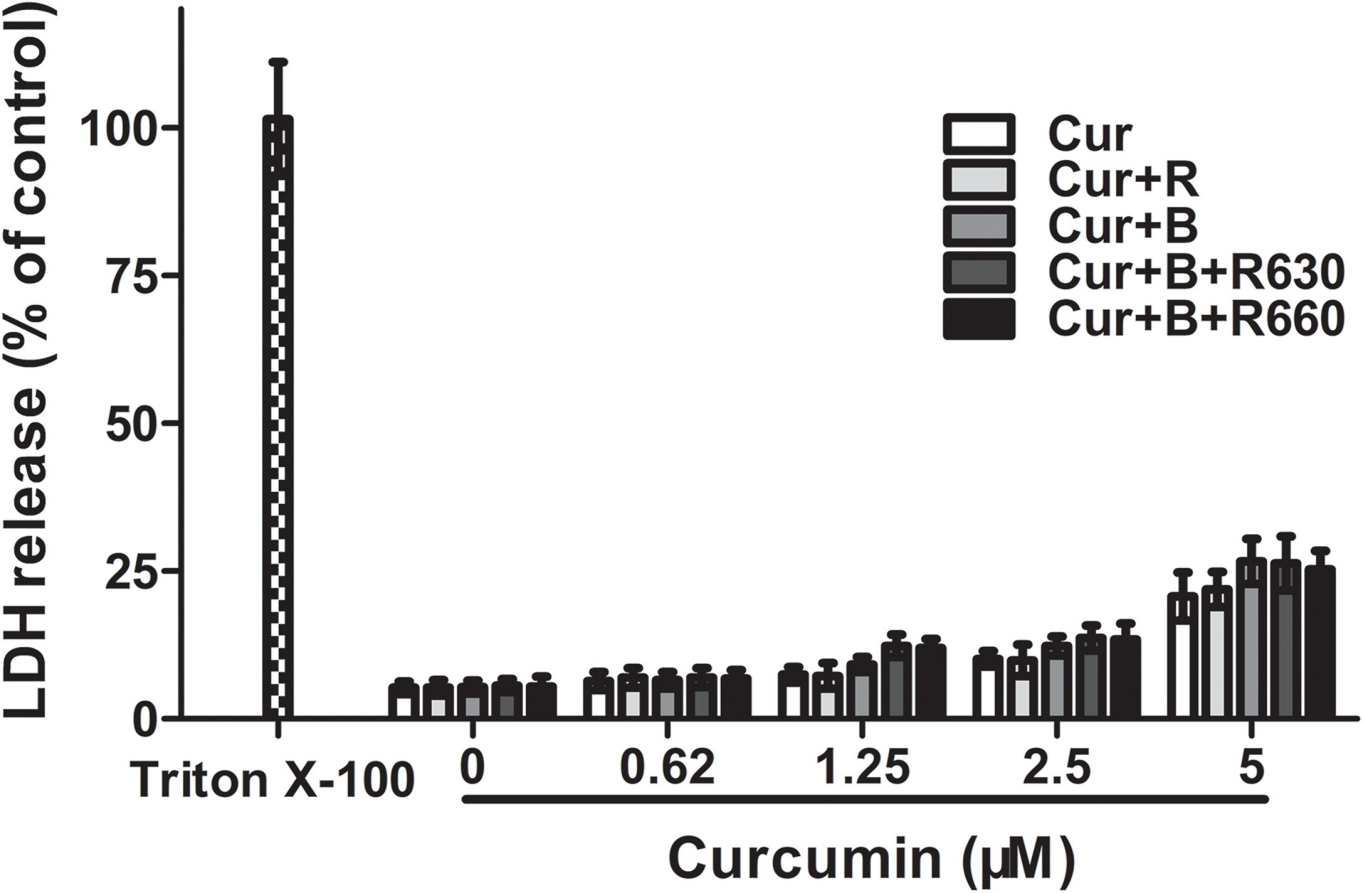
Curcumin combined with red united blue light did not induce toxic membrane damage. HaCaT cells were pre-incubated with curcumin (0–5 μM) for 2 h, and then separately irradiated with blue light, red light and two combinations of blue and red light, or protected from light. The lactate dehydrogenase (LDH) concentration in the cell-free supernatants was measured 20 h later. Cells treated with 1% Triton X-100 served as a positive control. All values were referred to the positive control. The assessment was implemented by three independent experiments.

Features of necrotic cell death include the loss of cell membrane integrity, cell swelling, dissolved and the release of inflammatory cytoplasmic components [22]. To explore whether the observed proliferation-inhibiting effect of curcumin in the presence of red united blue light leads to toxic membrane injury, we inspected the cytosolic levels of the lactate dehydrogenase (LDH) that was released into culture medium. We pre-incubated HaCaT cells with different concentrations of curcumin (0–5 μM) for 2 h and then irradiated the cells with blue light, red light and two combinations of blue and red light. Twenty hours later, the release of lactate dehydrogenase in the cell supernatants was measured. The positive control cells were treated with 1% Triton X-100, and the other cells were treated as stated above. The results exhibited that no prominent curcumin concentration dependent liberation of LDH was observed. As shown in Fig 3, the red light or blue light irradiation alone did not augment the release of LDH compared with controls. As well, the integrity of the cell membrane was neither altered by curcumin alone nor by a combination of curcumin (0.16–5 μM) and blue added to red light irradiation. These results clearly showed that the treatment using curcumin combined with light irradiation reduced the cell viability of HaCaT cells but the integrity of the cell membrane was still remained.

### Curcumin combined with red united blue light induced apoptosis in HaCaT cells

**Fig 4:**
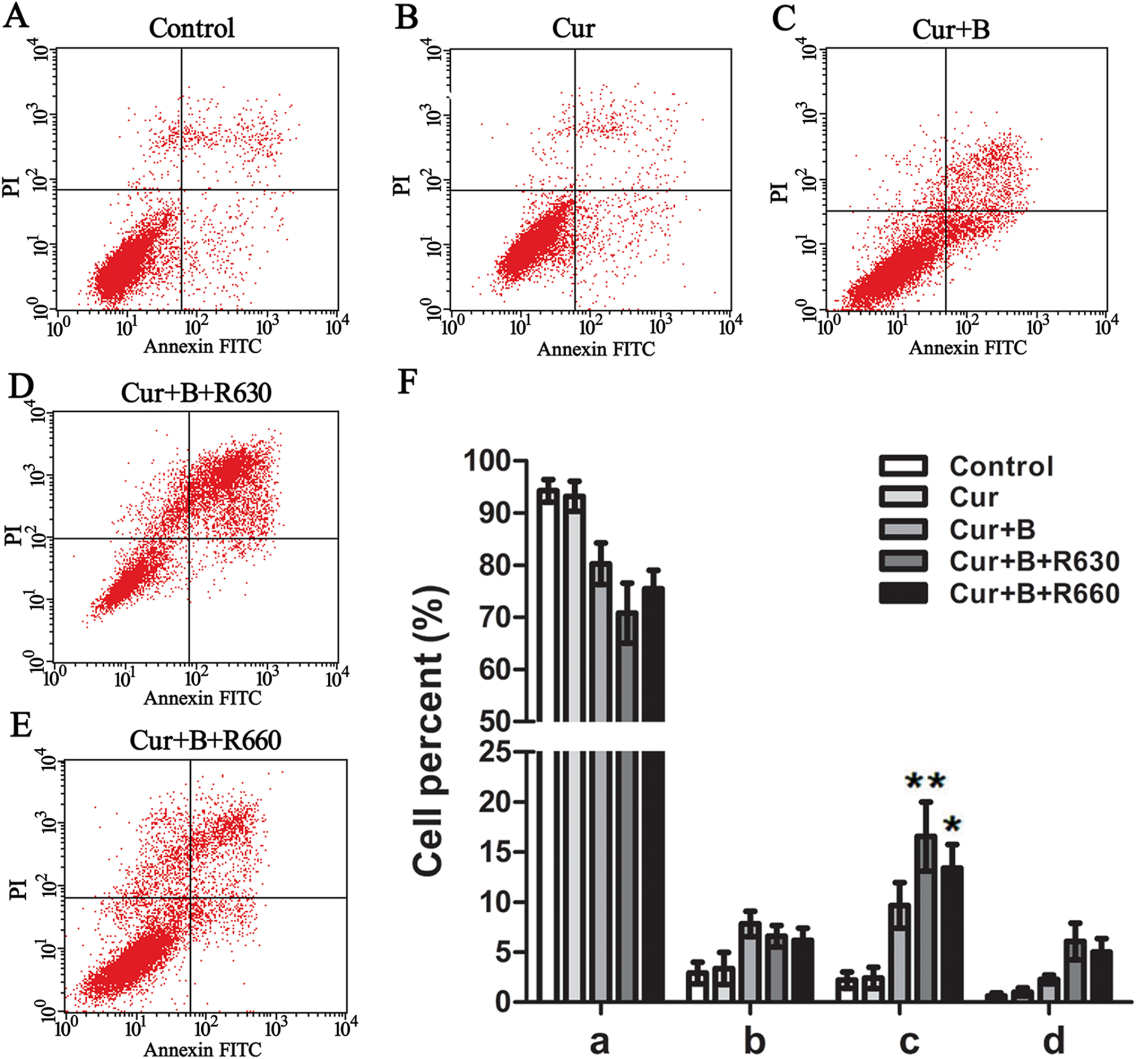
Curcumin combined with red united blue light induced apoptosis in HaCaT cells. (A) Flow cytometric analysis of HaCaT cells without any treatment. (B)-(E) Flow cytometric analysis of HaCaT cells which were pre-incubated with curcumin (2.5 μM) for 2 h and then protected from light or separately irradiated with blue light and two combinations of blue and red light.(F) The apoptotic rate of cells was measured by the percentage of early apoptotic cells added late apoptotic cells. a, b, c and d represent normal cells, early apoptotic cells, late apoptotic cells and dead cells, respectively. Bars with different characters are statistically different at *p*<0.05(*) or *p*<0.01(**) level. All images shown are representative of three independent experiments.

Suppression of cell proliferation could be induced by cell apoptosis or cell cycle arrest or an association of both of these two ways. To investigate the underlying mechanism how curcumin combined red united blue light irradiation generates cell death, we detected the apoptotic rate of cells using a flow cytometric Annexin V assay. HaCaT cells were pre-treated with 2.5 μM curcumin for 2 h and then irradiated with blue light, red light or two combinations of blue and red light. After twenty hours, the cell apoptosis distribution was determined with a typical series of flow cytometry histograms (Fig 4). In the cells which were treated with curcumin and protected from light or the cells which were irradiated with red light or blue light alone, there was no significant increase in the percentage of apoptotic cells (shown in S2 Fig). The combined treatment of curcumin and blue light increased the number of apoptotic cells, but the effect was not obvious (*p>*0.05). The combination of blue united red light and curcumin treatment led to a conspicuous increase in the percentage of apoptotic cells, especially cells in late stage apoptosis, as shown in Fig 4. The extent of the effects was different for cells treated with curcumin in combination with blue added red light at 660 nm (*p<*0.05) and cells treated with the combination of blue and red light at 630 nm (*p<*0.01), which indicated that the combined treatment with curcumin and blue united red light at 630 nm attained more significant apoptosis inducing effect. To investigate whether the combination of red united blue light and curcumin triggers apoptosis, we also strained cells with bisbenzimide to observe the formation of apoptotic bodies as well as the nuclear morphology. Our results showed cells treated with curcumin and blue light united red light irradiation had more cell rounding and more apoptotic bodies than cells treated with curcumin alone or light irradiation alone.

### Curcumin combined with red united blue light inhibited cell proliferation

**Fig 5:**
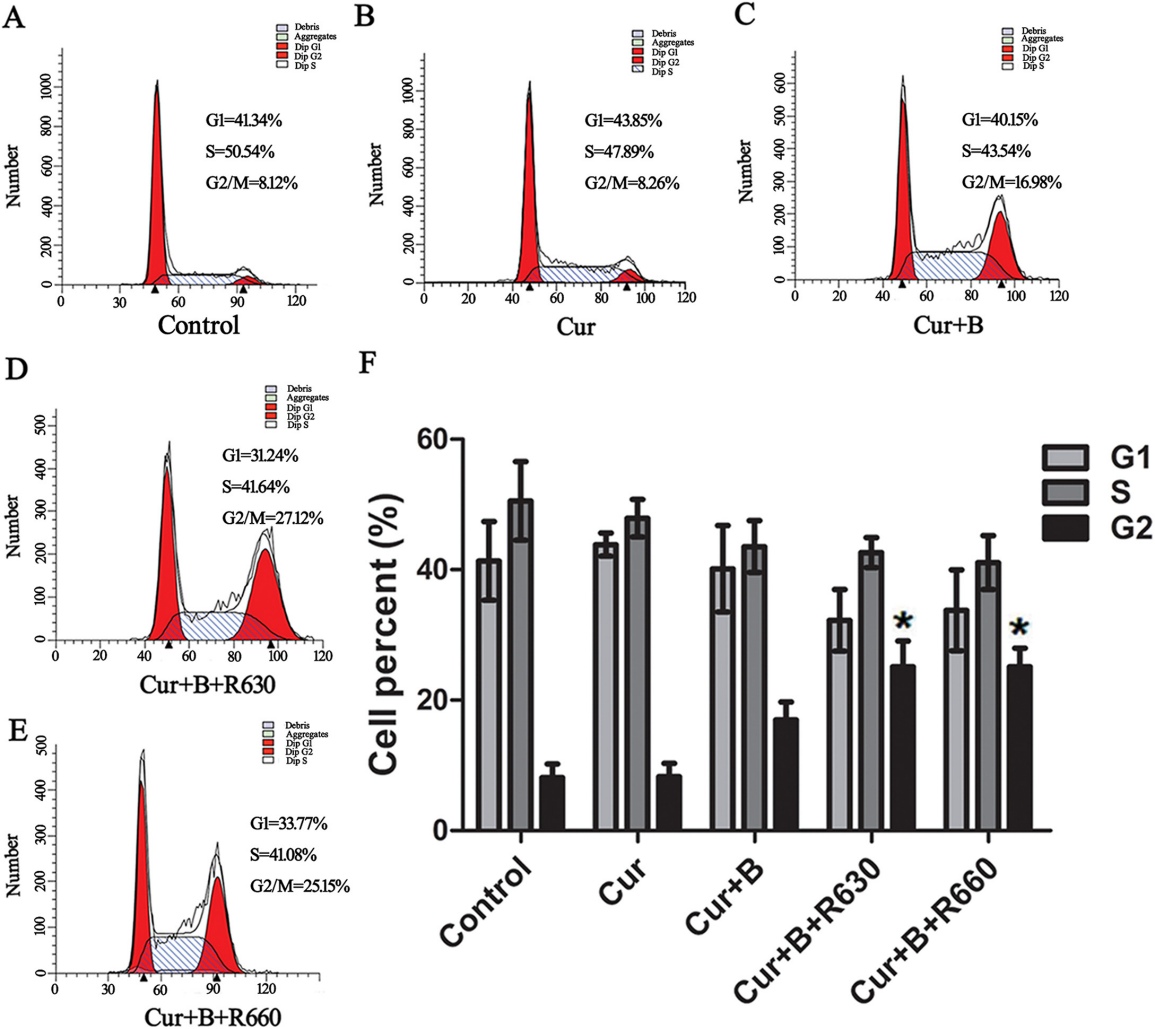
Curcumin combined with red united blue light inhibited cell proliferation. (A) Flow cytometric analysis of HaCaT cells without any treatment. (B)-(E) Flow cytometric analysis of HaCaT cells which were pre-incubated with curcumin (2.5 μM) for 2 h and then protected from light or separately irradiated with blue light and two combinations of blue and red light. (F) Quantification of cell cycle distribution (G1, S and G2/M). Each bar represents the mean of the three independent experiments, and the differences between cells treated with light irradiation or not are particularly evident at *p*<0.05(*) or *p*<0.01(**) level.

At the same time, we also detected the distribution of cell cycles via flow cytometric analysis. HaCaT cells were pre-treated with 2.5 μM curcumin for 2 h and then irradiated with blue light, red light or two combinations of blue and red light. Twenty hours later, the cell cycle distribution was determined with a typical series of flow cytometry histograms (Fig 5). The measured data did not change between the control groups and light irradiated alone groups (shown in S3 Fig). As shown in Fig 5, irradiation of curcumintreated cells with blue light had some cell proliferation inhibition in HaCaT cells, but the effect was not remarkable (*p>*0.05). However, irradiation of curcumin-treated cells with blue added red light led to distinct cell proliferation inhibition in HaCaT cells, and our experiments demonstrated that the cell cycle was arrested specially at the G2/M transition point (*p<*0.05). These results suggested that inhibition of proliferation occured after treatment with low doses of curcumin combined with red united blue light irradiation.

### Curcumin combined with red united blue light restrained TNF-α-induced NF-κB activation

**Fig 6:**
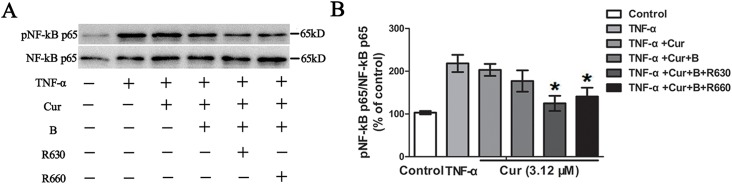
Curcumin combined with red united blue light inhibited TNF-α-induced NF-κB activation. HaCaT cells were pre-incubated with curcumin (3.12 μM) for 2 h, and then separately irradiated with blue light and two combinations of blue and red light, or protected from light. Subsequently, the cells were treated with TNF-α (20 ng/ml) for 1 h, and the nuclear extracts were prepared and analysed. (A) The expression level of phospho NF-κB p65 (pNF-κB p65) was detected by western blot, with NF-κB p65 as a loading control. (B) Densitometry analysis of phosphorylated p65. Bars with different characters are statistically different at *p*<0.05(*) level. The results shown are representative of three independent experiments.

Considering NF-κB plays an important role in the cellular stress, such as inflammation, apoptosis inhibition and cell proliferation, we also investigated the effect of curcumin combined with red united blue light on TNF-α-induced NF-κB activation. HaCaT cells were pre-treated with 3.12 μM curcumin for 2 h and then irradiated with blue light, red light or two combinations of blue and red light. Following irradiation, cells were treated with 20 ng/ml TNF-α for 1 h. As indicated by western blot in which the nuclear extracts from TNF-α-stimulated cells were incubated with the antibody against the p65 (RelA) subunit of NF-κB and phospho p65, TNF-α induced p65 NF-κB activation was inhibited by the combination of curcumin and blue light, but the difference was not evident (*p*>0.05). When the cells were treated with blue united red light, the results were dramatically more significant (*p*<0.05), which was demonstrated by the decreased migration of phosphorylated p65 NF-κB (Fig 6) and p65 NF-κB into the nucleus (S4 Fig). However, the light irradiation alone did not alter the transference of phosphorylated p65 NF-κB (shown in S5 Fig). Our results suggested that TNF-α promoted NF-κB activation in HaCaT cells, whereas curcumin combined with blue added red light dramatically inhibited that activity. The inhibition of NF-κB may accelerate the apoptosis and enhance cell cycle arrest of HaCaT cells.

### Curcumin combined with red united blue light induced caspase activation

**Fig 7:**
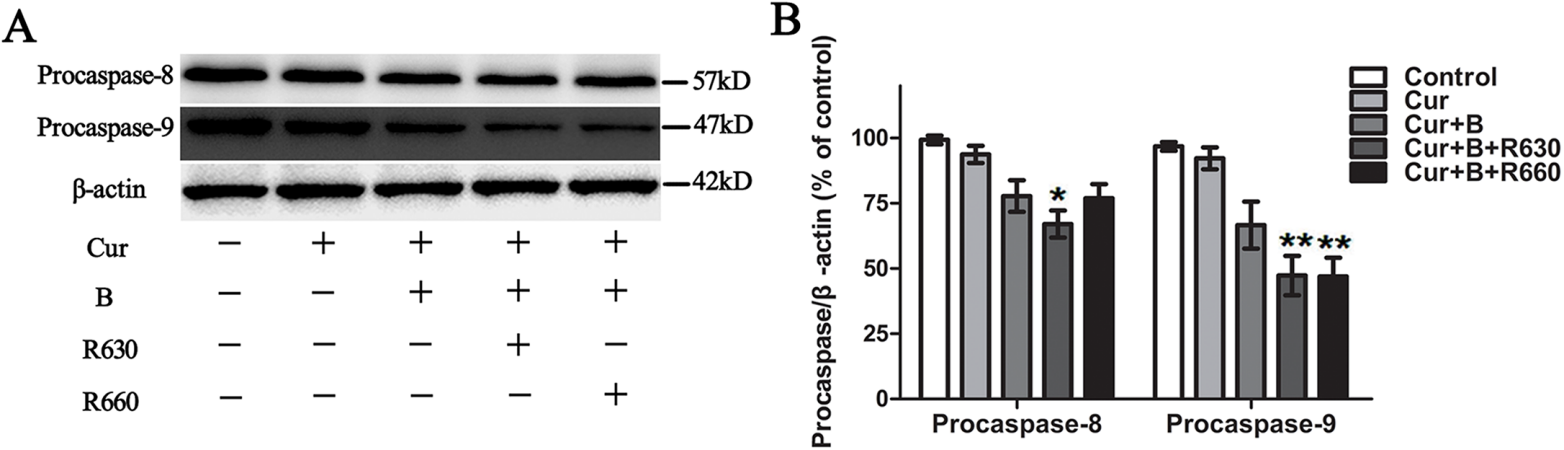
Curcumin combined with red united blue light induced caspase activation. HaCaT cells were pre-incubated with curcumin (2.5 μM) for 2 h, and then separately irradiated with blue light and two combinations of blue and red light, or protected from light. Twenty hours after the combined treatments, the lysates were prepared and analysed. (A) The expression level of inactive forms of caspase-8 and caspase-9 (procasepase-8 and procaspase-9) were inspected by western blot, with β-actin as a loading control. (B) Densitometry analysis of procasepase-8 and procaspase-9. Bars with different characters are statistically different at *p*<0.05(*) or *p*<0.01(**) level. Each bar represents the mean of three independent experiments.

In order to further clarify the induction of cell apoptosis, we examined the caspase activity as an early indicator. HaCaT cells were pre-treated with 2.5 μM curcumin for 2 h and then irradiated with blue light, red light or two combinations of blue and red light. Twenty hours after the combined treatment, the lysates were analysed by western blot using specific antibodies against caspase-9 and caspase-8, respectively. Our results showed that curcumin together with blue light induced weak activation of caspase-9 and caspase-8, but the effect was not evident (*p*>0.05). However, curcumin combined with blue united red light at 630 nm had an even more remarkable effect on the activation of caspase-9 (*p*<0.01) and caspase-8 (*p*<0.05), respectively; nonetheless, curcumin combined with blue united red light at 660 nm only promoted an obvious activation of caspase-9 (*p*<0.05), but had particularly feeble influence on the activation of caspase-8 (*p*>0.05), which was proved through evidence of the cleavage of inactived full-length procaspase-9 and procaspase-8 (Fig 7). In comparison, single curcumin treated cells or light alone irradiated cells showed no difference in the activation of caspase-8 and caspase-9 compared to controls, and similarly, curcumin combined with red light irradiation also had little effect (shown in S6 Fig and Fig 7).

### Curcumin combined with red united blue light restrained TNF-α-activated MAPKs and Akt

**Fig 8:**
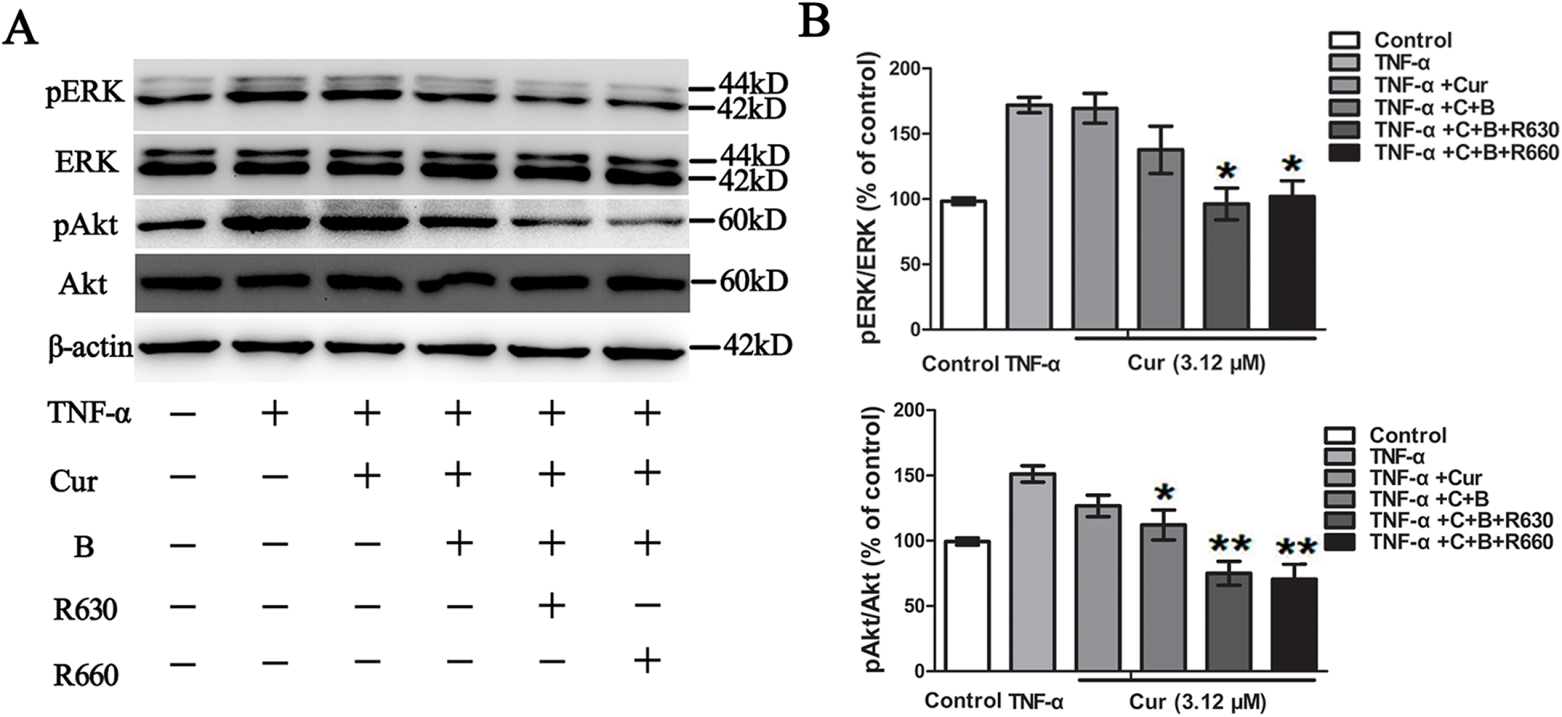
Curcumin combined with red united blue light restrained TNF-α-activated ERK and PKB/Akt. HaCaT cells were pre-incubated with curcumin (3.12 μM) for 2 h, and then separately irradiated with blue light and two combinations of blue and red light, or protected from light. Subsequently, the cells were treated with TNF-α (20 ng/ml) for 2 h, and the whole cell lysates were prepared and analysed. (A) The phosphorylation levels of ERK and Akt were measured by western blot, with total ERK and Akt served as loading controls. (B) Densitometry analysis of pERK and pAkt. Bars with different characters are statistically different at *p*<0.05(*) or *p*<0.01(**) level. The results shown are representative of three independent experiments.

Considering the intrinsic apoptosis pathway is not only associated with MAPK signalling pathway but also linked to PI3/PKB, we also investigated the impact of curcumin in combination with blue united red light on the extracellular regulated protein kinases ERK and growth associated kinases PKB/Akt. HaCaT cells were pre-treated with 3.12 μM curcumin for 2 h and then irradiated with blue light, red light or two combinations of blue and red light. Following the irradiation, the cells were treated with 20 ng/ml TNF-α for 2 h. Our results suggested that TNF-α promoted ERK and Akt activation in HaCaT cells. Curcumin combined with blue light had a feeble inhibition effect on the phosphorylation of ERK, but the function was not evident (*p*>0.05); whereas curcumin combined with blue united red light dramatically inhibited the activation of ERK (*p*<0.05), which was indicated by western blot analysis (Fig 8). However, curcumin in combination with blue light irradiation exhibited an apparent effect on phosphorylation level of Akt (*p*<0.05); meanwhile, curcumin combined with blue united red light observably reduced the activation of Akt (*p*<0.01). As shown in Fig 8, phosphorylation of ERK and Akt induced by TNF-α was markedly decreased in curcumin combined with and blue united red light treated HaCaT cells, which implied that curcumin combined with blue united red light attenuated TNF-α-induced ERK and Akt activation in HaCaT cells. In curcumin alone treated groups, red light or blue light irradiated alone groups, these effects were not observed (S7 Fig). These results revealed that the apoptosis induced by curcumin combined with blue united red light irradiation also included the down regulation of ERK and Akt phosphorylation.

## Discussion

Recently, PDT has appeared as a hopeful therapeutic tool for the treatment of dermatologic conditions, such as psoriasis and superficial basal cell carcinoma [10, 23]. Several reports have proved that PDT simultaneously assisted with a photosensitizer can induce diverse cellular responses, which increases the accumulation in the target cells and leads to induction of cell necrosis or apoptosis [9, 14].

Curcumin is a highly polyphenic molecule that was originally found to display antibacterial activity in 1949 [24]. Since then, curcumin has become more and more striking due to its anti-inflammatory, anti-oxidation, anti-proliferative, apoptosis-promoting and radiosensitive properties [25–27]. Curcumin has been verified to be safe, tolerated and non-toxic, even at a high dose up to 8 g per day [28]. The proliferation inhibition and apoptosis inducing effects of curcumin have been documented sufficiently in many different cells, which indicates that curcumin could be used for treating hyper-proliferative diseases [17]. However, the effective usage of curcumin is impeded by its particularly low absorption via trans-dermal or oral bioavailability and rapid systemic elimination, which seriously hinders the curative effect of curcumin [15, 16, 29]. Therefore, approaches to strengthen and improve the bioavailability of curcumin are favourable. Curcumin has a rather wider absorption bands range from 300 nm to 500 nm [13], and curcumin combined with UV or visible light irradiation acquires the maximum light absorption at about 420 nm [30, 31]. By adding auxiliary of visible light irradiation, the cytotoxicity of curcumin is enhanced [17, 32, 33].

For the most of effective activation of curcumin, the maximum absorption of the skin should equally be considered for the optimal treatment potency. Blue light without the accretion of exogenous photosensitizers also has an inherent anti-proliferation effect and exhibits fewer cytotoxic effects in mammalian cells compared with the ultraviolet irradiation. For instance, irradiation with blue light at 400–420 nm only at high dosages, shows toxic and side effects compared with the ultraviolet irradiation [6]. The effectiveness of blue light in treating hyper-proliferative skin attributes to its ability to delay proliferation [6]. Additionally, a lot of blue light-emitting lamps are available with a maximum emission wavelength range from 400 nm to 440 nm [7].

Because of the deeper light penetration into the skin, red light is widely preferred in PDT [7, 8, 34]. In addition, red light may also exert anti-inflammatory effects via regulating the release of inflammatory factors. However, the definite regulative action underlying the advantageous effects of red light is yet not totally understood [35, 36]. In previous acne studies, mixed LED red and blue light therapy was proved to be more efficient treatment than blue light alone, which may due to the combination of the anti-bacterial and anti-inflammatory characteristics of the light [37].

Therefore, the aim of this study was to assess the effectiveness of the combination of low concentrations of curcumin and red mixed blue light on treating hyper-proliferative skin conditions.

In the present study, we used LED arrays with a distinct wavelength of 405 nm combined with low concentrations of curcumin (0.62–3.12 μM), and observed a reduction in the proliferative capacity of HaCaT cells. The inhibition of cell proliferation was strongly enhanced when cells were treated with blue combined with red light at 630 nm or 660 nm. Our studies also demonstrated that HaCaT cells were arrested at a specific transition point, particularly at the G2/M transition point, when they were treated with a low concentration of curcumin (2.5 μM) combined with blue light added red light irradiation. In the control groups, neither a low concentration of curcumin alone nor single light irradiation induced a visible inhibition in cell proliferation. These results suggested that not only the photo-catalytic effect attribute of curcumin but also the photo-activation is essential when taking advantage of curcumin at low concentrations. These findings were agreed with previous results [14, 17].

One of the most remarkable features of curcumin is the apoptosis-inducing effect. In this study, we observed that the combined treatment of curcumin and blue united red light irradiation triggered apoptosis in HaCaT cells, which was indicated by the activation of caspase-8 and caspase-9, the formation of apoptotic bodies, the inhibition of NF-κB activation and the down-regulation of phosphorylated ERK and Akt, but did not destroy the integrity of cell membrane. Our results distinctly proved that the combination of curcumin and blue united red light irradiation might be a very effective approach for regulating proliferation and apoptosis in skin keratinocytes.

Compared to treatment with curcumin alone or single light irradiation, significant decreases of procaspase-9 were detected after utilizing a combination of a sub-apoptotic concentration of curcumin at 2.5 μM with blue light united red light irradiation. However, only the combination of curcumin with blue united red light at 630 nm induced an evident reduction of procaspase-8. When treated with curcumin combined with blue united red light irradiation, the apoptosis-inducing effect was obviously enhanced, suggesting that apoptosis is efficiently motivated by coinstantaneous treatment with curcumin and blue united red light through both caspase-8 and caspase-9 activation. Previous studies have reported that the photosensitizer effect of curcumin was enhanced by the irradiation of UVB in HaCaT cells [10] and the the cytotoxicity of curcumin was strengthened by irradiation with visible light in nasopharyngeal cancer cell lines [32]. A conceivable molecular mechanism of the photo-toxicity of curcumin might be that curcumin photo-generates reduced forms of molecular oxygen [38]. Caspase activation is an early sign of apoptosis. Two principal pathways involved in cell apoptosis are the mitochondrial-mediated (intrinsic) pathway and the death receptor-mediated (extrinsic) pathway [39, 40]. Casepase-9 is a dominating initiator in the intrinsic pathway mediated by mitochondria. On the contrary, caspase-8 is a principal initiator of the extrinsic apoptotic pathway regulated by the death receptors.

Simultaneously, these results were further verified by flow cytometric test consequences. Treatment with curcumin and blue united red light irradiation led to a conspicuous increase in the proportion of apoptotic cells, especially in late stage apoptosis, which may sensitize cells to apoptosis via activating caspase pathways. And the red light at 630 nm had an even intense effect than that at 660 nm. Treating cells with curcumin and blue light also exhibited some effects on inducing apoptosis, but the difference was not significant. Taken together, these results demonstrated that both the intrinsic and extrinsic apoptosis pathways were involved in the apoptosis induced by curcumin and blue united red light irradiation in HaCaT cells.

NF-κB is a nuclear transcription factor that mediates a large number of gene expression, which are critical for the regulation of apoptosis, inflammation and multifarious autoimmune diseases [41]. It is well known that high concentration of curcumin is a potential inhibitor of the NF-κB transcription factor in various human cells [42, 43]. Our results displayed that low concentrations of curcumin combined with blue united red light apparently inhibited TNF-α-induced NF-κB activation. Consistent with previous reports, the restraint of NF-κB activation expedited apoptosis and cell cycle arrest in our study.

Intracellular MAPK signalling pathway plays a vital role in the regulation of cell proliferation and cell apoptosis [44]. In addition to activating caspasemedicated apoptosis and NF-κB activation, TNF-α is also known to activate MAPKs. ERK, one of the most important MAPK pathways, could hinder cell apoptosis through obstructing caspase activation [37, 45]. Our results showed that a low concentration of curcumin or single light irradiation had little effect on the phosphorylation level of ERK. However, when curcumin was combined with blue light united red light irradiation, TNF-α-induced activation of ERK was significantly attenuated. These results indicated that the decline of phosphorylation level of ERK may facilitate the activation of caspases, which played a vital role in promoting apoptosis.

In addition, the Akt pathway also plays an important role in cell apoptosis and cell proliferation regulation [46]. It has reported that Akt could increase the cell viability via suppressing the expression of pro-apoptotic proteins [47] and medicate the expression and activation of NF-κB [48]. Our results showed that curcumin combined with blue united red light irradiation significantly decreased the TNF-α-induced activation of Akt. Nevertheless, curcumin alone or single light irradiation nearly exhibited no effect on the activation of Akt pathway. These results suggested that curcumin combined with blue united red light irradiation may also induce apoptosis via suppressing the activation of Akt.

Based on above consequences, we proposed that light irradiation enhanced the cellular absorption of curcumin, especially the combined usage of blue and red light which is likely to integrate the anti-bacterial and anti-inflammatory characteristics of the light, efficiently compensating the trouble of the low bioavailability of curcumin. The combined treatment with curcumin and blue united red light irradiation significantly inhibited TNF-α-induced activation of Akt and ERK signaling pathways, which cause mitochondrial dysfunction and the inhibition of NF-κB activity.

Mitochondrial dysfunction and the inhibition of NF-κB activity lead to liberation of apoptosis factors, and then activate both the intrinsic and extrinsic apoptosis pathways, and finally result in amplified curcumin-induced cell apoptosis and cell growth arrest in HaCaT cells.

## Conclusions

Our present results clearly demonstrated that the strategy to combine curcumin and blue light united red light irradiation could be a useful and highly efficient method for enhancing the anti-hyperproliferative activities of curcumin. It was noteworthy that blue light united red light irradiation, which combined the anti-proliferative and anti-inflammatory activities to maximize the stimulation of the target photosensitizer and reached the photodynamic target spot located in the deep dermis, showed to be more efficient than blue light alone. Taking advantage of different wavelength under treatment of hyper-proliferative illness will perfect treatment effect and simultaneously alleviate adverse side reactions.

## Supporting Information

S1 Fig The effect of curcumin and light irradiation on cell viability of HaCaT cells. The effect of the combination of curcumin and light irradiation on cell viability of HaCaT cells (n = 3).
(TIF)

S2 Fig The effects of light irradiation on HaCaT cell apoptotic death. (A) Flow cytometric analysis of HaCaT cells which were protected from light or separately irradiated with red light and blue light. (B) The apoptotic rate of cells was measured by the percentage of early apoptotic cells added late apoptotic cells. All images shown are representative of three independent experiments.
(TIF)

S3 Fig Flow cytometric analysis of HaCaT cell treated with light irradiation. HaCaT cells were irradiated with blue light or red light, or light protected as described above. As shown in the figure: (A) light-protected control; irradiated with blue light; irradiated with red light at 630 nm; irradiated with irradiated with red light at 660 nm. (B) Quantification of cell cycle distribution (G1, S and G2/M).
(TIF)

S4 Fig The effect of curcumin combined with red united blue light on TNF-α-induced NF-κB subunit p65 expression. HaCaT cells were pre-incubated with curcumin (3.12 μM) for 2 h, and then separately irradiated with blue light and two combinations of blue and red light, or protected from light. Subsequently, the cells were treated with TNF-α (20 ng/ml) for 1 h, and the nuclear extracts were prepared and analysed. (A) The expression level of p65 was detected by western blot, with Histone H3 as a loading control. (B) Densitometry analysis of p65. Bars with different characters are statistically different at *p*<0.05(*) level.
(TIF)

S5 Fig The effects of light irrdiaiton on TNF-α-induced NF-κB activation. HaCaT cells were irradiated with blue light, red light or light protected respectively, as described in materials and methods. Subsequently, the cells were treated with TNF-α (20 ng/ml) for 1 h and the nuclear extracts were prepared and analysed. (A) The expression level of p65 was detected by western blot, with Histone H3 as a loading control. (B) Densitometry analysis.
(TIF)

S6 Fig The effects of light irradiation on HaCaT cell caspase activation. HaCaT cells were irradiated with blue light, red light or light protected, respectively, as described above. Twenty hours after the combined treatment, the lysates were prepared and analysed. (A) The level of inactive forms of caspase-8 and caspase-9 (procasepase-8 and procaspase-9) were detected by western blot, with β-actin as a loading control. (B) Densitometry analysis of procasepase-8 and procaspase-9.
(TIF)

S7 Fig The effects of light irradiation on TNF-α-induced activation of ERK and PKB/Akt. HaCaT cells were irradiated with blue light, red light or light protected respectively. Then, the cells were treated by TNF-α (20 ng/ml) for 2 h, and the whole cell lysates were prepared and analysed. (A) The phosphorylation levels of ERK and Akt were measured by western blot, with total ERK and Akt served as loading controls. (B) Densitometry analysis of pERK and pAkt.
(TIF)
