# Supplementary figures and images for: Red Light Combined with Blue Light Irradiation Regulates Proliferation and Apoptosis in Skin Keratinocytes in Combination with Low Concentrations of Curcumin
Source: PLoS One. 2015 Sep 18;10(9):e0138754. doi: 10.1371/journal.pone.0138754 (PMC4575166; doi:10.1371/journal.pone.0138754)

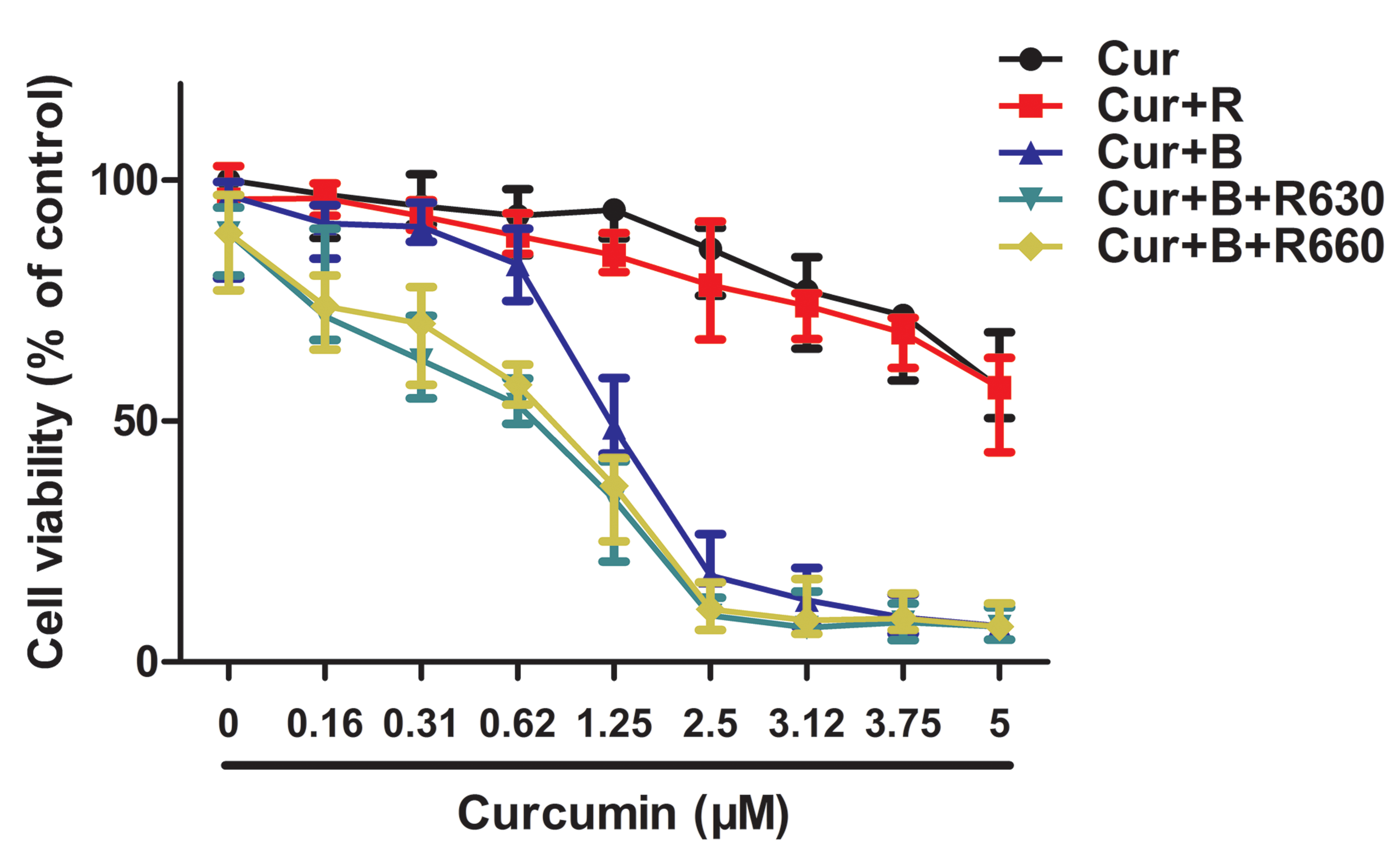

Supplement: S1 Fig — The effect of the combination of curcumin and light irradiation on cell viability of HaCaT cells (n = 3). (TIF) [file pone.0138754.s001.tif]

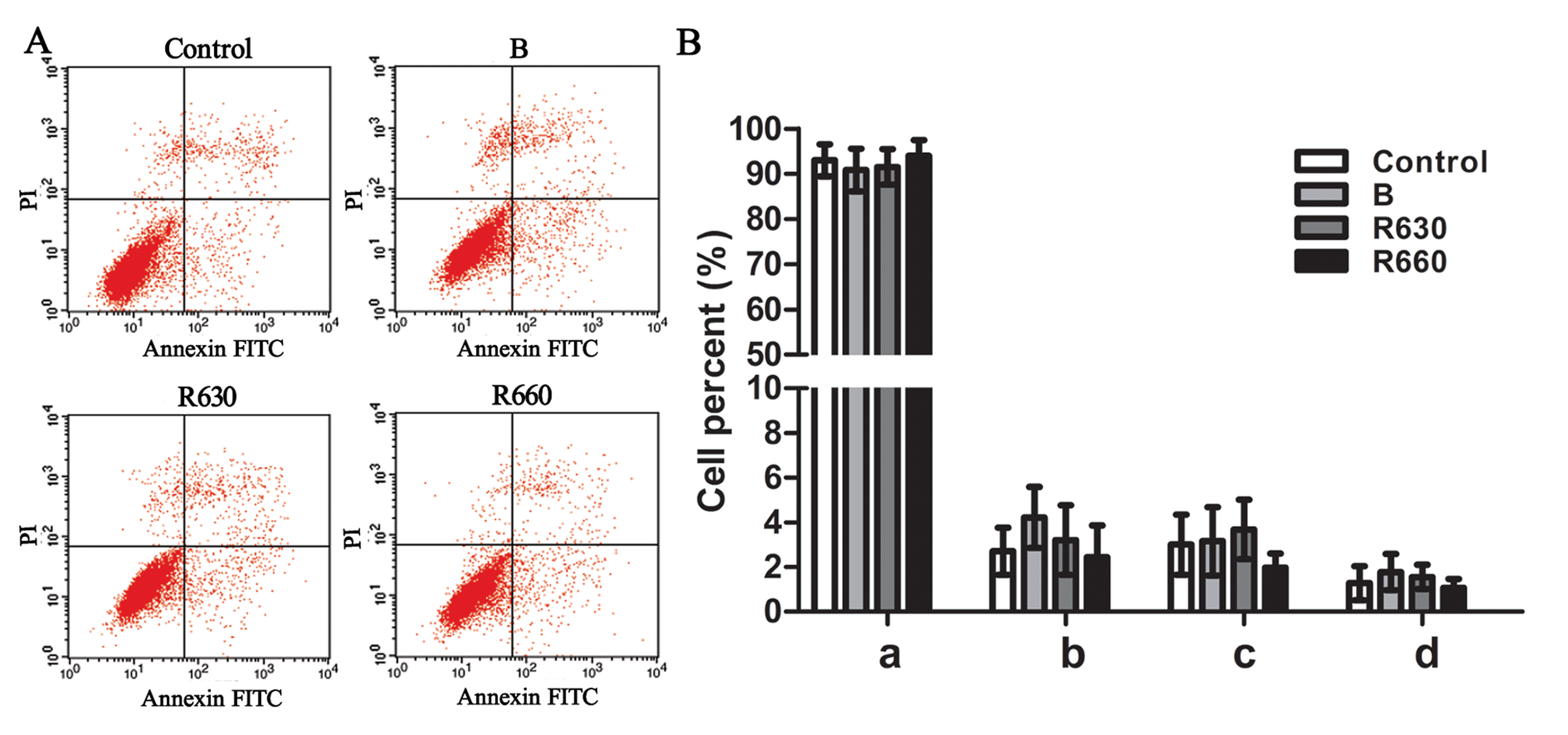

Supplement: S2 Fig — (A) Flow cytometric analysis of HaCaT cells which were protected from light or separately irradiated with red light and blue light. (B) The apoptotic rate of cells was measured by the percentage of early apoptotic cells added late apoptotic cells. All images shown are representative of three independent experiments. (TIF) [file pone.0138754.s002.tif]

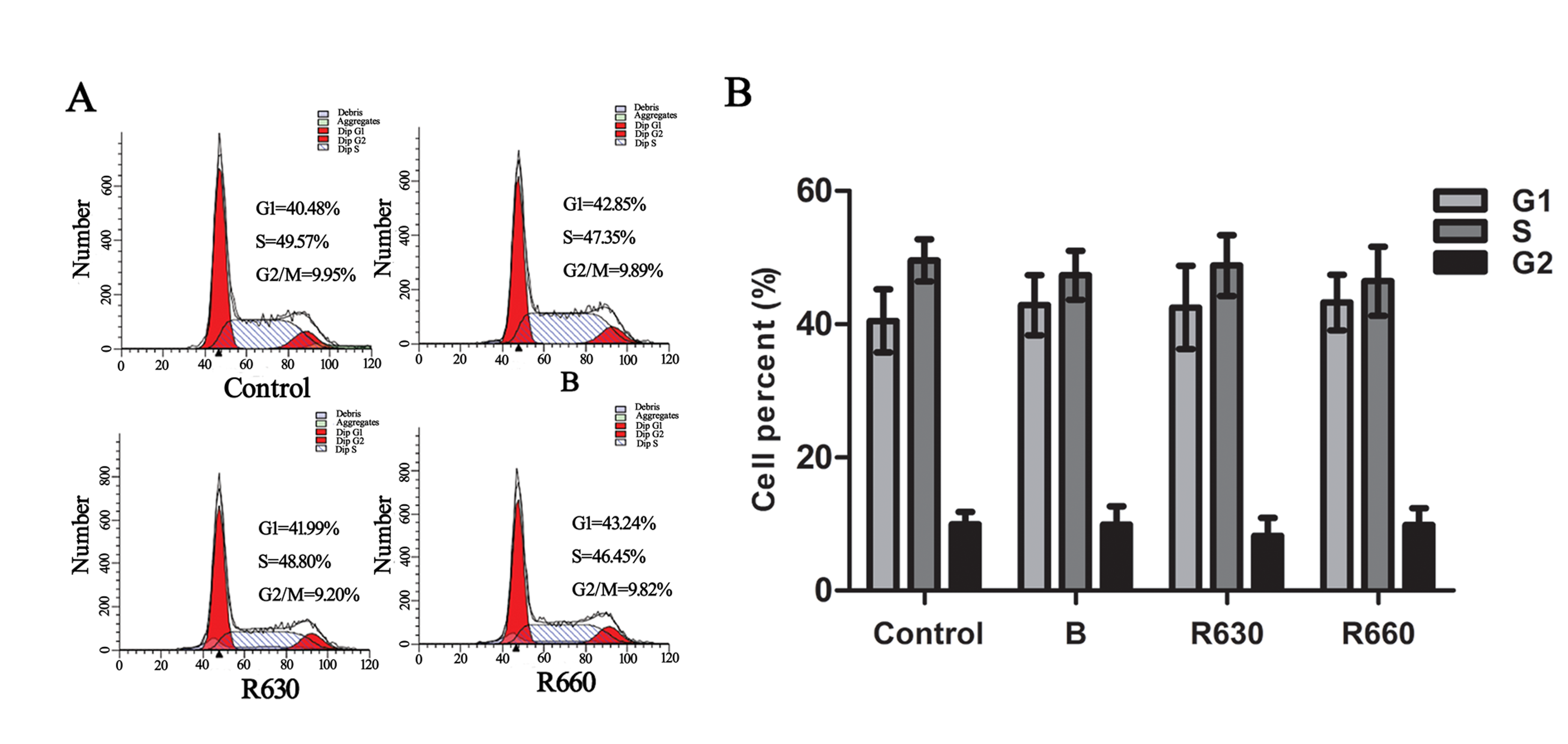

Supplement: S3 Fig — HaCaT cells were irradiated with blue light or red light, or light protected as described above. As shown in the figure: (A) light-protected control; irradiated with blue light; irradiated with red light at 630 nm; irradiated with irradiated with red light at 660 nm. (B) Quantification of cell cycle distribution (G1, S and G2/M). (TIF) [file pone.0138754.s003.tif]

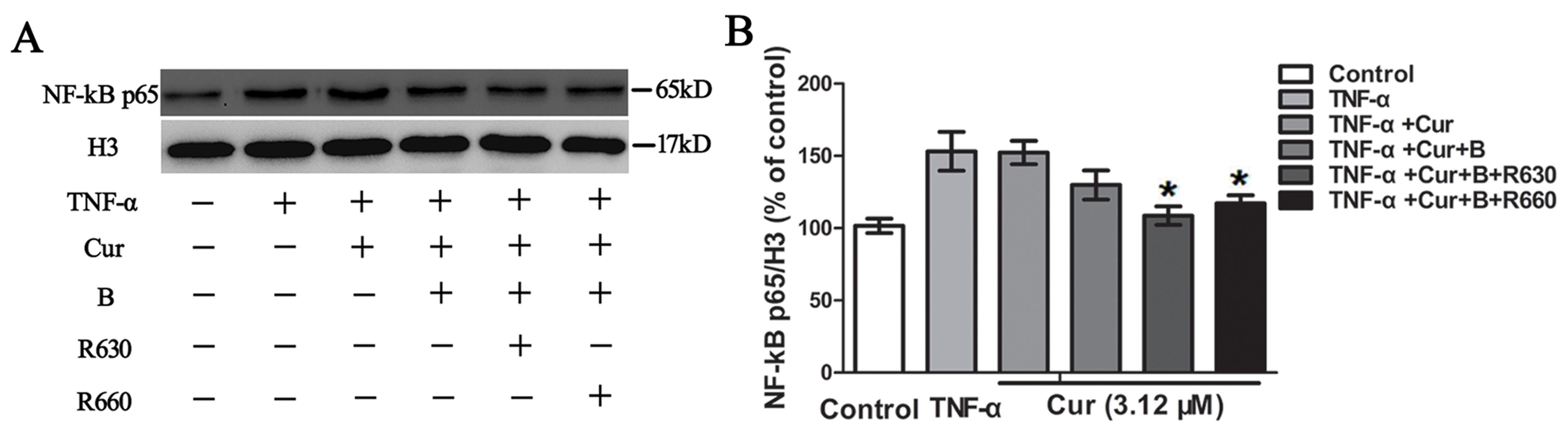

Supplement: S4 Fig — HaCaT cells were pre-incubated with curcumin (3.12 μM) for 2 h, and then separately irradiated with blue light and two combinations of blue and red light, or protected from light. Subsequently, the cells were treated with TNF-α (20 ng/ml) for 1 h, and the nuclear extracts were prepared and analysed. (A) The expression level of p65 was detected by western blot, with Histone H3 as a loading control. (B) Densitometry analysis of p65. Bars with different characters are statistically different at p<0.05(*) level. (TIF) [file pone.0138754.s004.tif]

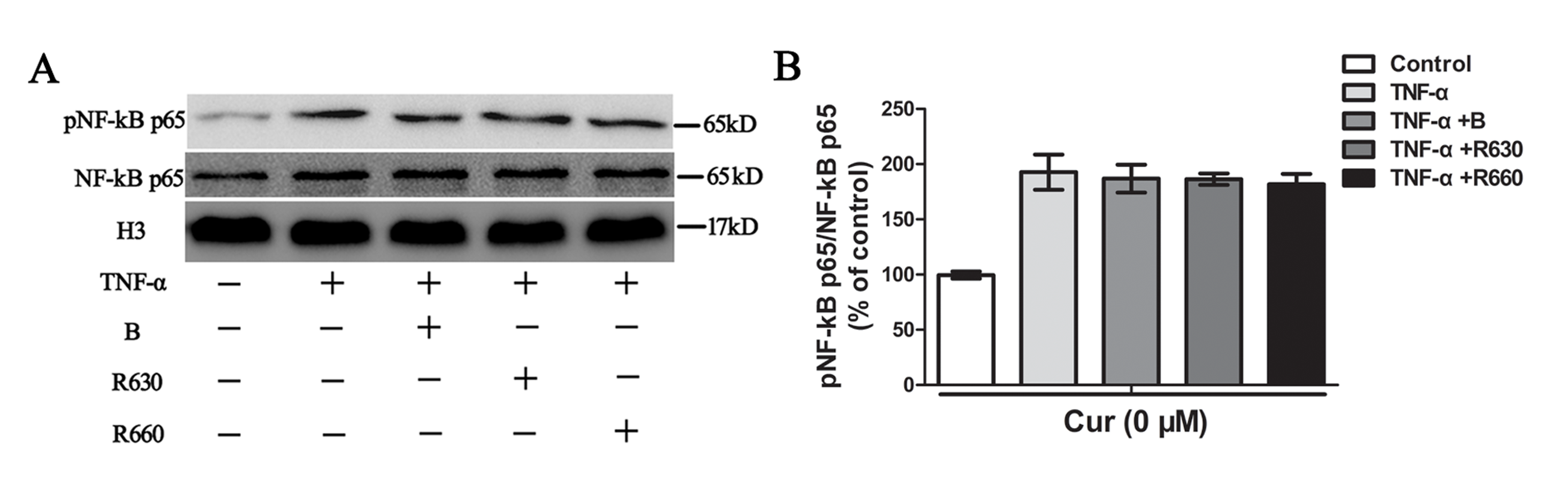

Supplement: S5 Fig — HaCaT cells were irradiated with blue light, red light or light protected respectively, as described in materials and methods. Subsequently, the cells were treated with TNF-α (20 ng/ml) for 1 h and the nuclear extracts were prepared and analysed. (A) The expression level of p65 was detected by western blot, with Histone H3 as a loading control. (B) Densitometry analysis. (TIF) [file pone.0138754.s005.tif]

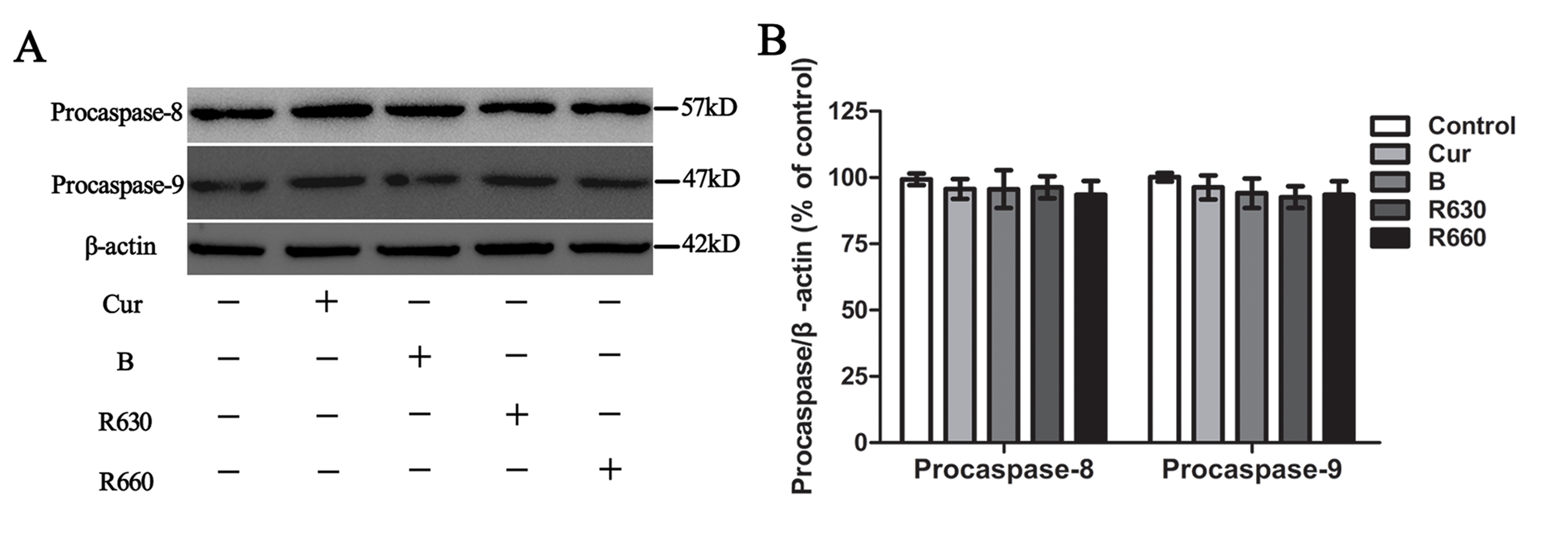

Supplement: S6 Fig — HaCaT cells were irradiated with blue light, red light or light protected, respectively, as described above. Twenty hours after the combined treatment, the lysates were prepared and analysed. (A) The level of inactive forms of caspase-8 and caspase-9 (procasepase-8 and procaspase-9) were detected by western blot, with β-actin as a loading control. (B) Densitometry analysis of procasepase-8 and procaspase-9. (TIF) [file pone.0138754.s006.tif]

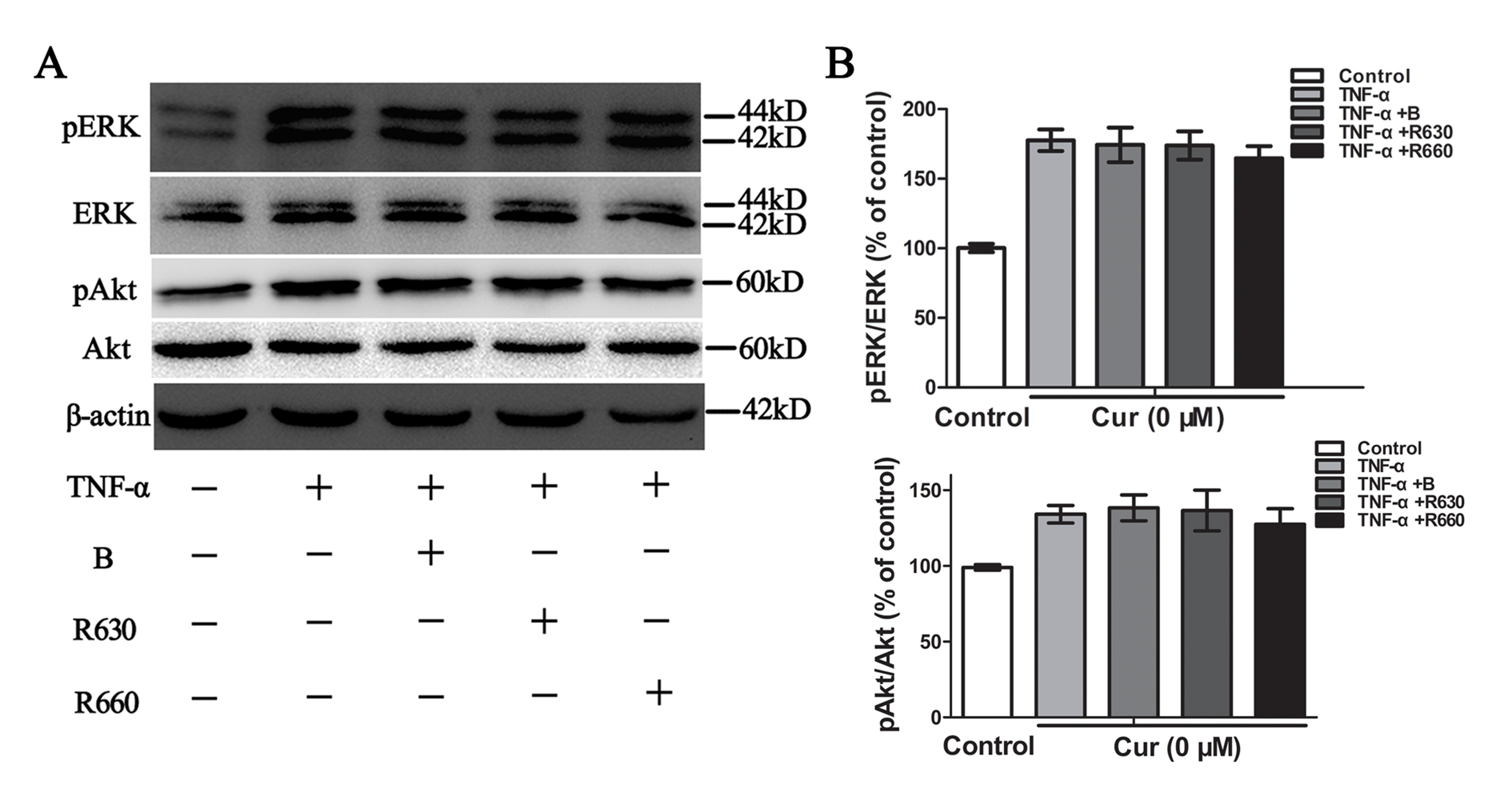

Supplement: S7 Fig — HaCaT cells were irradiated with blue light, red light or light protected respectively. Then, the cells were treated by TNF-α (20 ng/ml) for 2 h, and the whole cell lysates were prepared and analysed. (A) The phosphorylation levels of ERK and Akt were measured by western blot, with total ERK and Akt served as loading controls. (B) Densitometry analysis of pERK and pAkt. (TIF) [file pone.0138754.s007.tif]
